# Correction: Lin et al. Effects of Nitric Oxide on Bladder Detrusor Overactivity Through the NRF2 and HIF-1α Pathways: A Rat Model Induced by Metabolic Syndrome and Ovarian Hormone Deficiency. *Int. J. Mol. Sci.* 2024, *25*, 11103

**DOI:** 10.3390/ijms27021045

**Published:** 2026-01-21

**Authors:** Hung-Yu Lin, Jian-He Lu, Rong-Jyh Lin, Kuang-Shun Chueh, Tai-Jui Juan, Jing-Wen Mao, Yi-Chen Lee, Shu-Mien Chuang, Mei-Chen Shen, Ting-Wei Sun, Yung-Shun Juan

**Affiliations:** 1 School of Medicine, College of Medicine, I-Shou University, Kaohsiung 84001, Taiwan; ed100464@edah.org.tw; 2 Division of Urology, Department of Surgery, E-Da Cancer Hospital, I-Shou University, Kaohsiung 824005, Taiwan; 3 Division of Urology, Department of Surgery, E-Da Hospital, I-Shou University, Kaohsiung 82445, Taiwan; 4 Center for Agricultural, Forestry, Fishery, Livestock and Aquaculture Carbon Emission Inventory and Emerging Compounds, General Research Service Center, National Pingtung University of Science and Technology, Pingtung County 912301, Taiwan; toddherpuma@mail.npust.edu.tw; 5 Department of Parasitology, School of Medicine, College of Medicine, Kaohsiung Medical University, Kaohsiung 807378, Taiwan; rjlin@kmu.edu.tw; 6 Graduate Institute of Clinical Medicine, College of Medicine, Kaohsiung Medical University, Kaohsiung 807378, Taiwan; spacejason69@yahoo.com.tw; 7 Department of Urology, Kaohsiung Municipal Ta-Tung Hospital, Kaohsiung 80661, Taiwan; 8 Department of Urology, Kaohsiung Medical University Hospital, Kaohsiung 80756, Taiwan; blast2337@gmail.com (J.-W.M.); u9181002@gmail.com (S.-M.C.); bear5824@gmail.com (M.-C.S.); ting.wei0220@gmail.com (T.-W.S.); 9 Kaohsiung Armed Forces General Hospital, Kaohsiung 802301, Taiwan; terryjuan@gmail.com; 10 Department of Thoracic Surgery Division, Kaohsiung Veterans General Hospital, Kaohsiung 813414, Taiwan; 11 Department of Anatomy, School of Medicine, College of Medicine, Kaohsiung Medical University, Kaohsiung 807378, Taiwan; yichen83@kmu.edu.tw; 12 Department of Urology, College of Medicine, Kaohsiung Medical University, Kaohsiung 807378, Taiwan

**Error in Figure**

In the original publication [1], inadvertent errors occurred during the preparation of several figure panels, including Figure 2A (OVX + MetS group, micturition pressure analysis), Figure 2B (MetS + L-arginine group, voiding frequency analysis), Figure 3A (MetS + L-NAME group and OVX + MetS group, electrical field stimulation analysis), Figure 5C (MetS + L-arginine group, immunostaining of E-cadherin), Figure 5G (MetS + OVX + L-NAME group, immunostaining of E-cadherin), Figure 7H (Western blot of M3), and Figure 9A (Western blot of NDUFS3). Specifically, some image panels were inadvertently duplicated or misplaced during figure assembly. The affected panels have now been carefully re-examined and replaced with the correct images derived from the original experimental data. These corrections involve only the presentation of the figures and do not affect the experimental results, statistical analyses, or the scientific conclusions of the article. The corrected Figure 2, Figure 3, Figure 5, Figure 7 and Figure 9 appear below. The authors confirm that the scientific conclusions are unaffected. This correction was approved by the Academic Editor. The original publication has also been updated.

## Figures and Tables

**Figure 2:**
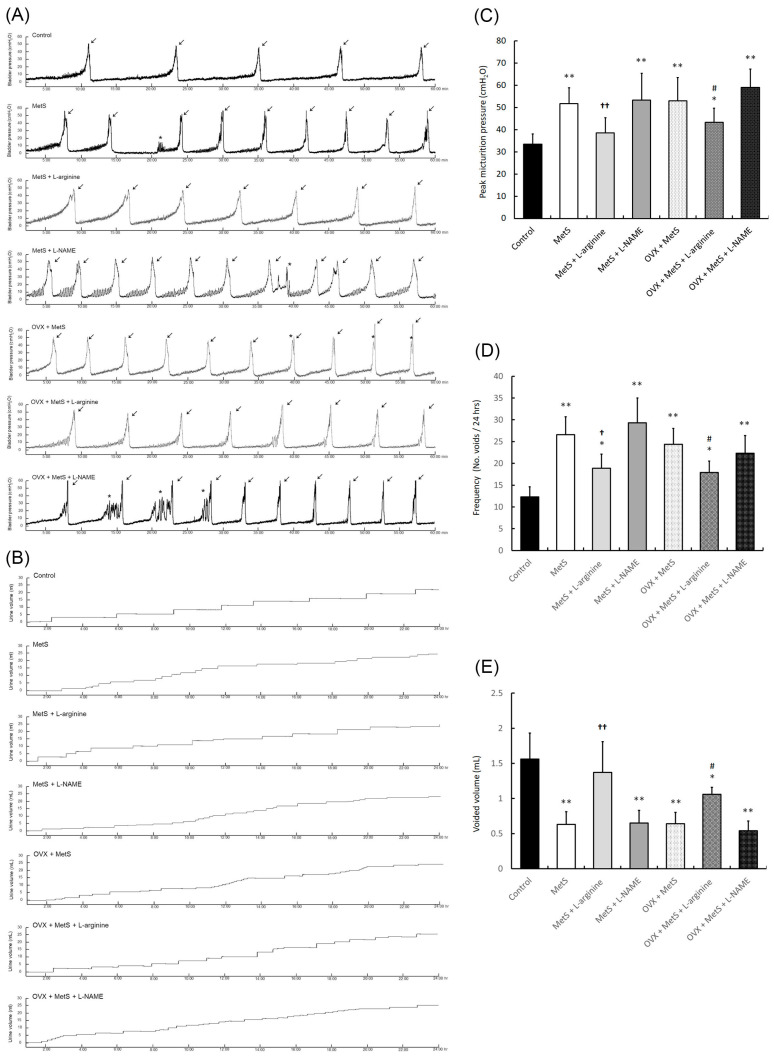
L-arginine improved voiding behavior and alleviated detrusor hyperactivity in a rat model. Urodynamic analysis of cystometric parameters (**A**), including micturition pressure (**A**,**C**), voiding frequency, contraction (arrows), and non-voiding contraction (asterisks), in the different groups. Tracing analysis of 24-h voiding behavior by metabolic cage, including voiding frequency (**B**,**D**) and volume (**B**,**E**) in the different groups. The MetS + OVX group exhibited increased bladder micturition pressure, voiding contractions, non-voiding contractions, and micturition frequency, whereas the L-arginine groups showed an improved bladder voiding pattern and volume. Note: MetS, metabolic syndrome; OHD, ovarian hormone deficiency. Data were expressed as mean ± SD for *n* = 6. * *p* < 0.05; ** *p* < 0.01 versus the control group. ^†^ *p* < 0.05; ^††^ *p* < 0.01 versus the MetS group. ^#^ *p* < 0.05 versus the MetS + OVX group.

**Figure 3:**
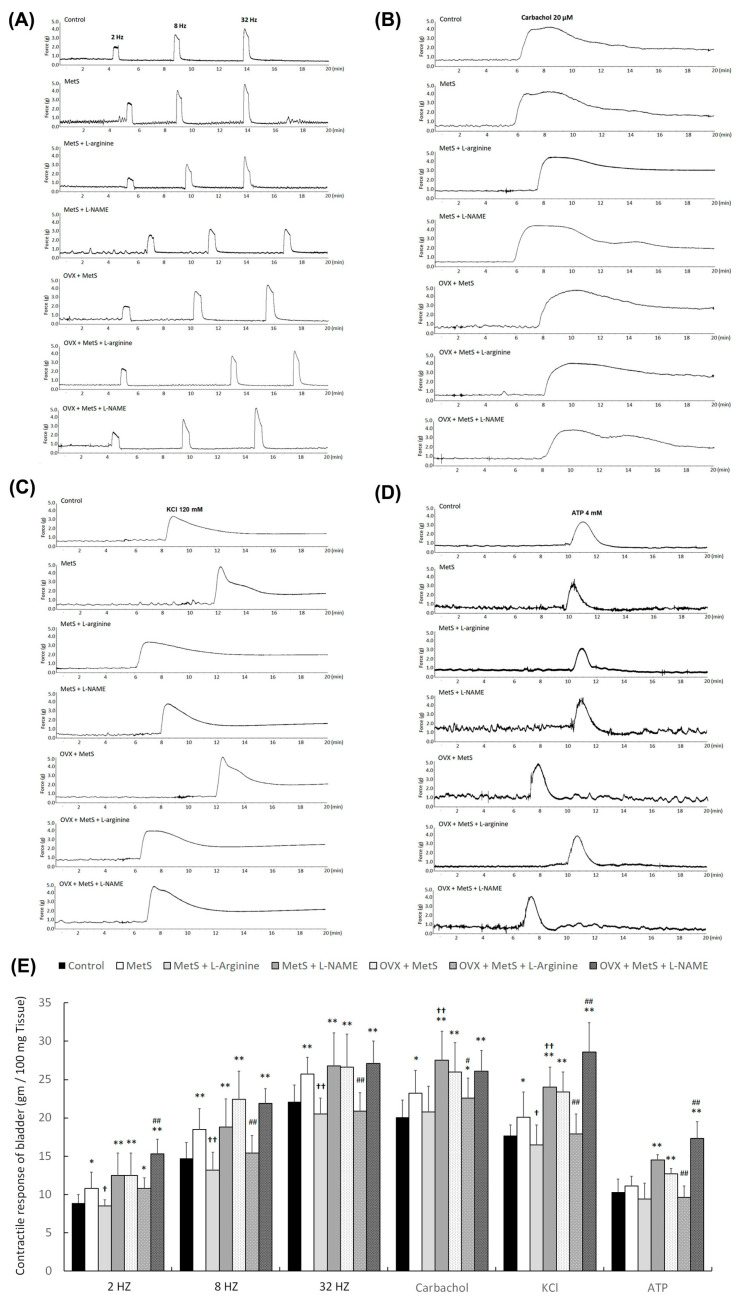
L-arginine treatment improved the bladder detrusor contractile response. After 12-month HFHS feeding with or without OVX, bladder strips induced by EFS (**A**,**E**), carbachol (**B**,**E**), KCl (**C**,**E**), and ATP (**D**,**E**) in the MetS group, the MetS + L-NAME group, the MetS + OVX group, and the MetS + OVX + L-NAME group, they had higher contractile responses compared with the control group, whereas the MetS + L-arginine and MetS + OVX + L-arginine groups demonstrated significantly lower contractile responses compared to the MetS and MetS + OVX groups. L-arginine treatment significantly ameliorated the detrusor contractile response to various forms of stimulation in the MetS + L-arginine group and the MetS + OVX + L-arginine group. Note: EFS, electrical field stimulation; OVX, bilateral ovariectomy. Data were expressed as mean ± SD for *n* = 6. * *p* < 0.05; ** *p* < 0.01 versus the control group. ^†^ *p* < 0.05; ^††^ *p* < 0.01 versus the MetS group. ^#^ *p* < 0.05; ^##^ *p* < 0.01 versus the MetS + OVX group.

**Figure 5:**
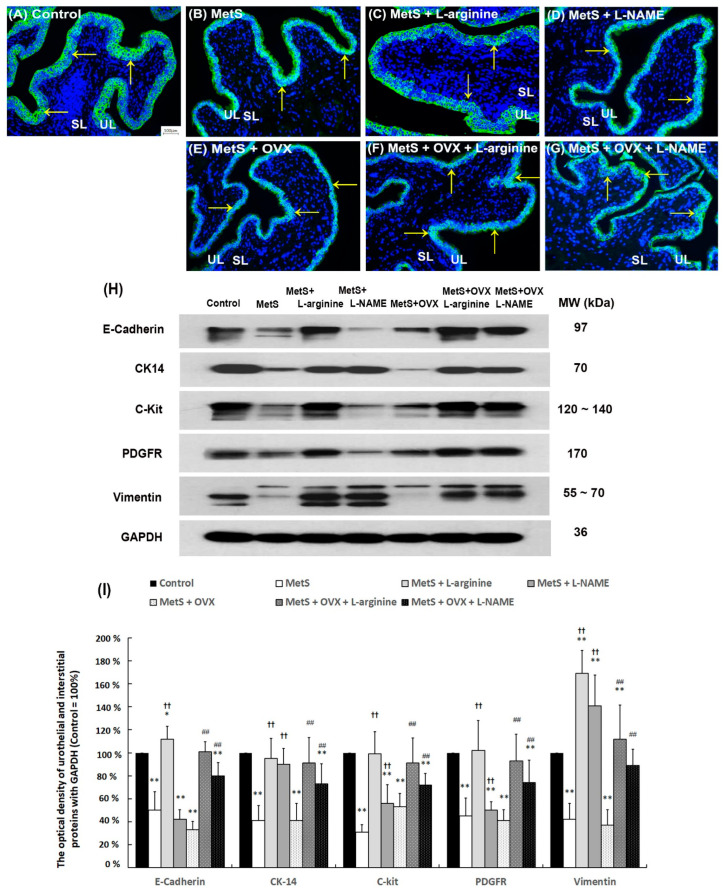
L-arginine improved bladder urothelial regeneration and interstitial cell generation. In a rat model of MetS with or without OHD-induced detrusor hyperactivity, urothelial marker (E-Cadherin), cell proliferating proteins (CK14), and IC markers (C-Kit, vimentin, and PDGFR) were quantified by immunostaining (**A**–**G**) and Western Blots (**H**,**I**). (**A**–**G**): In the control group (**A**), E-Cadherin staining showed the urothelial layer (UL; yellow arrows) consisting of three to five layers. However, following a HFHS diet with or without OVX, the bladders displayed a thinner and defective urothelial mucosa in the UL. Morphological evaluation in the MetS + L-arginine group (**C**) and the MetS + OVX + L-arginine group (**F**) showed an increased thicker layer of UL to improve bladder damage induced by MetS with or without OHD. (**A**–**G**) magnification × 400; Scale bar (grey) = 100 μm. (**H**,**I**): Western Blot analysis of E-Cadherin, CK14, C-Kit, vimentin, and PDGFR expressions was investigated. All expressions in the MetS group and the MetS + OVX group were significantly declined as compared with the control group, whereas all expressions in the MetS + L-arginine group and the MetS + OVX + L-arginine group were significantly enhanced compared to the MetS group and the MetS + OVX group. Results were normalized as the control = 100%. Note: IC, interstitial cell; MetS, metabolic syndrome; ML, muscular layer; UL, urothelial layer; SL, suburothelial layer; OVX, bilateral ovariectomy; OHD, ovarian hormone deficiency. Data were expressed as mean ± SD for *n* = 6. * *p* < 0.05; ** *p* < 0.01 versus the control group. ^††^ *p* < 0.01 versus the MetS group. ^##^ *p* < 0.01 versus the MetS + OVX group.

**Figure 7:**
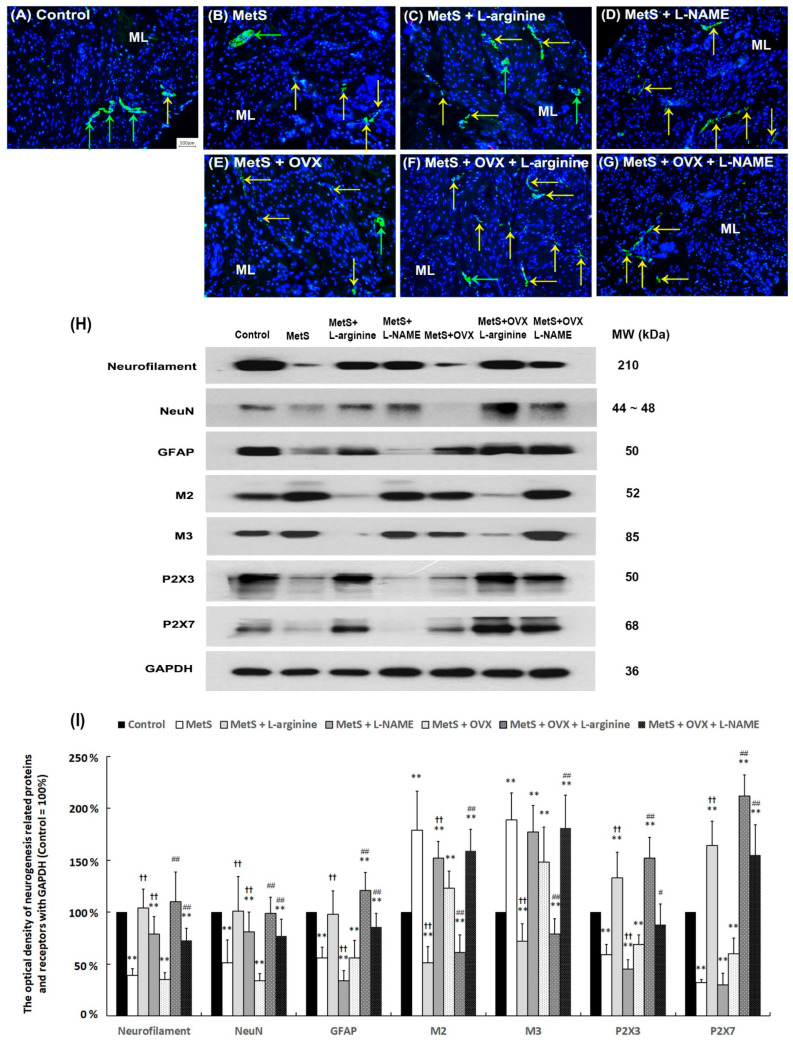
The effect of L-arginine increased neuronal regeneration, synaptic transmission, and receptor response. The expressions of neuronal endogenous markers (neurofilament, NeuN, GFAP), muscarinic receptor (M2 and M3) markers, and purinergic receptor (P2X3 and P2X7) markers were assessed by immunostaining (**A**–**H**) and Western Blots (**I**). (**A**–**G**): The distribution of neurofilament for neurogenesis was shown by immunostaining. Neurofilament immunostaining (yellow arrows) and ganglion (green arrows) were prominently expressed in the SL and ML of the control group (**A**). In contrast, the MetS group (**B**) and the MetS + OVX group (**E**) showed reduced neurofilament staining (yellow arrows) and ganglion (green arrows) in the thinner and defective urothelial mucosa of the SL and ML. However, neurofilament expression (yellow arrows) was significantly increased in the MetS + L-arginine group (**C**) and the MetS + OVX + L-arginine group (**F**) compared to the MetS group (**B**) and the MetS + OVX group (**E**). This indicates that L-arginine enhances bladder synaptic transmission, receptor response, and neurogenesis, thereby improving detrusor contractile. (**A**–**G**) magnification × 400; Scale bar (grey) = 100 μm. (**H**,**I**): Quantifications of the percentage of neurogenesis-related markers, muscarinic receptors, and purinergic receptors were evaluated by Western Blotting. Nuclear DNA was labeled with DAPI (blue). Note: NF, neurofilament; NeuN, neuronal nuclear antigen and neuron; GFAP, glial fibrillary acidic protein; ML, muscular layer. Results were normalized as the control = 100%. Data were expressed as mean ± SD for *n* = 8, ** *p* < 0.01 versus the sham group. ^#^ *p* < 0.05; ^##^ *p* < 0.01 versus the OVX group; ^††^ *p* < 0.01 versus the OVX + SW4 group.

**Figure 9:**
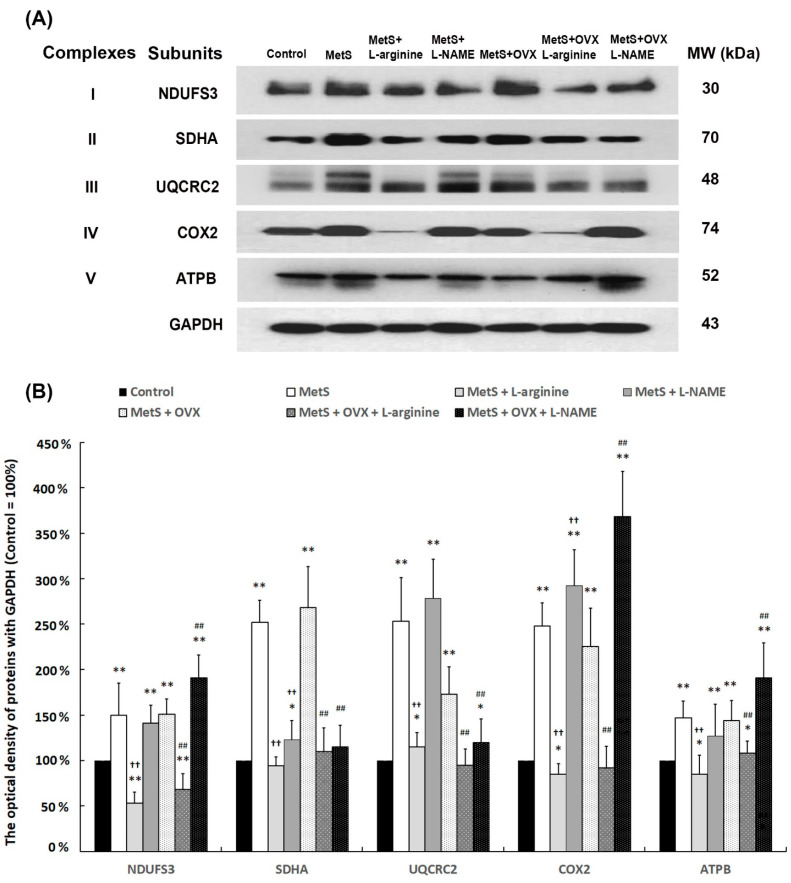
Up-regulation of the subunits of mitochondrial respiratory enzymes with MetS and OHD. (**A**) The expression levels of mitochondrial respiratory enzyme subunits (NDUFS3, SDHA, UQCRC1, COX-2, and ATPB) were analyzed by Western Blotting. (**B**) Quantification of these mitochondrial respiratory enzymes as a percentage relative to β-actin. Results were normalized to the control group, set at 100%. The expression levels of these subunits were elevated in the MetS group and significantly enhanced in both the MetS and MetS + OVX groups. Results were normalized as the control = 100%. Data were represented as mean ± SD for *n* = 6. * *p* < 0.05; ** *p* < 0.01 versus the control group. ^††^ *p* < 0.01 versus the MetS group. ^##^ *p* < 0.01 versus the MetS + OVX group.
